# Dual-Functional Polyurethane Sponge-Based Pressure Sensors Incorporating BZT/BTO, Polypyrrole, and Carbon Nanotubes with Energy Generation Capability

**DOI:** 10.3390/polym18020241

**Published:** 2026-01-16

**Authors:** Nurhan Onar Camlibel, Baljinder K. Kandola

**Affiliations:** 1 Textile Engineering Department, Engineering Faculty, Pamukkale University, Denizli 20020, Turkey; 2 Institute for Materials Research and Innovation, University of Greater Manchester, Deane Road, Bolton BL3 5AB, UK; b.kandola@greatermanchester.ac.uk

**Keywords:** pressure sensor, sponge, polypyrrole, carbon nanotube, sol-gel, energy generation

## Abstract

Flexible and wearable pressure sensors are essential for monitoring of human motion and are distinguished by their increased sensitivity and outstanding mechanical robustness. In this study, we systematically engineered a flexible and wearable pressure sensor with a multilayer conductive architecture, arranging a sponge substrate coated in a consecutive manner with a barium zirconium titanate thin film, followed by polypyrrole, multiwalled carbon nanotubes, and eventually polydimethylsiloxane. The foundation of additional conductive pathways is enabled via the utilization of a porous framework and the hierarchical arrangement, causing the achievement of an excellent sensitivity of 9.71 kPa^−1^ (0–9 kPa), a rapid 40 ms response time, and a fast 60 ms recovery period, combined with a particularly low detection limit (125 Pa) and an extended pressure range from 0 to 225 kPa. Furthermore, the integration of a rough and porous barium zirconium titanate/barium titanate thin film is expected to deliver a voltage output (1.25 V) through piezoelectric working mechanisms. This study possesses the potential to provide an innovative architecture design for advancing the development of future electronic devices for health and sports monitoring.

## 1. Introduction

Pressure sensors have been extensively investigated due to their capacity to transduce mechanical forces into electrical signals, making them a pivotal component in the forthcoming landscape of flexible and wearable electronic devices [1]. To date, the prevailing principles governing pressure sensors encompass alterations in capacitance or resistance induced by applied forces [2], as well as the mechanism of piezoelectric [3], triboelectric [4], piezoresistive [5], electrochemical, and ion-gradient-based systems [6]. Piezoresistive pressure sensor designs have been widely investigated in recent years for wearable and flexible sensing applications. Among previously reported studies, polyurethane sponges have found extensive utility as elastomeric substrates for the creation of conductive elastomeric composites by coating them with conductive polymers (polypyrrole (PPy), polyaniline (PANI), etc.), MXene, graphene, graphene oxide, and carbon nanotubes (CNTs). Their intricate, three-dimensional porous and reticular structure, capability of being folded and compressed, and fully interconnected network make them ideal for pressure sensor applications [7]. As the applied force increases, the conductive pathways expand, leading to a reduction in resistance due to an increase in transient contacts in the pressure sensors [8,9,10,11]. Wang et al. [12] have investigated a flexible polyurethane sponge pressure sensor modified with carbon black, multiwalled carbon nanotubes (MWCNTs), and thermoplastic polyurethane (TPU) to monitor human health status. They demonstrated that the sensor has high sensitivity (0.10 kPa^−1^ in a pressure range of 0–8 kPa) and fast response/recovery time (119/59 ms) together with good washability performance to detect human health status in real time. Conductive polymers, exemplified by PPy, exhibit attributes of softness and flexibility, thereby displaying favorable electrical conductivity, good adhesive characteristics, and a non-toxic nature, rendering them conductive materials [13]. Lv et al. prepared a polyurethane sponge piezoresistive sensor coated with graphene oxide and polypyrrole with a layer-by-layer assembly method [14]. The sponge sensors exhibited good sensitivity (0.79 kPa^−1^), a wide working range (75 Pa–15 kPa), excellent flexibility (85.5%), fast response time (70 ms), and outstanding cyclic stability (over 10,000 cycles). MWCNTs are commonly selected as exceptional conductive additives, owing to their high electrical conductivity and intricate three-dimensional architecture [15]. Wang et al. fabricated a CNT/polyurethane sponge piezoresistive pressure sensor through a dipping–drying method for human motion detection. The sensor displayed good sensitivity (2.7% kPa^−1^), fast response (response/recovery time of 60/100 ms), and excellent long-term stability [16]. Ma et al. developed MWCNT/reduced graphene oxide (rGO)@polyurethane (PU) sponge piezoresistive sensors with low densities (0.027–0.064 g cm^−3^), good compressibility (up to 75%), and high sensitivity [17]. Moreover, it was reported that the application of polydimethylsiloxane (PDMS) coating was employed to enhance the stability of the sensor and hinder the detachment of films and nanoparticles on the sponge surface [18]. A detailed literature review about employing polyurethane sponges, which are coated with PPy, PANI, carbon black, CNTs, graphite, MXene, PDMS, and barium titanate (BTO), as pressure sensors is given in Table S1 in the Supplementary Materials.

Furthermore, the demand for and interest in sustainable new energy sources have significantly increased due to the scarcity of present energy sources and their associated environmental issues. The development of hybrid wearable and flexible sensors that combine piezoresistive behavior with piezoelectric or triboelectric energy generation is highly relevant for sustainability and environmental impact as a new energy source, as it eliminates the need for batteries. Recently, piezoresistive pressure sensor designs have received significant attention for wearable and flexible sensors. However, only a limited number of studies have investigated piezoresistive pressure sensors with dual functionality enabled by piezoelectric or triboelectric mechanisms driven by human motion or other sources of mechanical energy, even though such strategies can supply energy for sensor operation. While piezoelectric and triboelectric devices can function as self-powered pressure sensors, their electrical outputs are generally transient and highly dependent on dynamic mechanical stimuli, limiting their effectiveness for continuous or static pressure detection. On the other hand, piezoresistive sensors yield stable and continuous signals but they typically require an external power source. The integration of piezoresistive sensing with piezoelectric or triboelectric components is therefore intended not to replace conventional sensing approaches, but rather to establish a dual-functional material platform that combines continuous pressure monitoring with mechanically induced electrical energy generation. Such hybrid systems provide enhanced functional versatility and represent a promising direction for wearable and flexible electronics. Ma et al. proposed a PPy@CNT@PU sponge-based triboelectric nanogenerator with an open-circuit voltage of 110 V and a short-circuit current of 12 µA [11]. Jo et al. coated PU sponges with MWCNT and poly(3,4-ethylenedioxythiophene) polystyrene sulfonate (PEDOT:PSS) to fabricate a triboelectric nanogenerator exhibiting good output performance and high durability [19]. Li et al. fabricated a ZnO@rGO@PU sponge piezoelectric nanogenerator backfilled with PDMS, achieving an open-circuit voltage of ~0.5 V and a short-circuit current density of ~2 µA/cm^2^ [20]. However, the piezoresistive sensing properties of the coated polyurethane foam were not characterized.

BTO is a perovskite material and has been comprehensively investigated as a piezoelectric material and a pressure sensor [3,21,22,23,24]. Barium zirconium titanate (BZT) has exhibited clear advantages over other piezoelectric materials such as ZnO and polyvinylidene fluoride (PVDF), including a higher piezoelectric coefficient, improved phase stability, a higher Curie temperature, and strong ferroelectric behavior. In addition, it is environmentally friendly and lead-free, offers a higher dielectric constant, possesses tunable properties through doping, and delivers superior energy generation performance. This compositional diversity inherently leads to adjustable piezoelectric characteristics [3,25]. Madbouly et al. prepared BTO/rGO nanocomposites by a co-precipitation method, and then polyurethane foam was coated with a chitosan solution and BTO/rGO nanocomposites. The obtained sensors displayed a high sensitivity of 2.64 kPa^−1^ and a good response time of 560 ms [3]. However, the energy generation performance of the coated polyurethane foam under mechanical stimuli was not characterized.

In this work, a composite material was deliberately designed by integrating components with complementary functionalities. The sponge serves as a compressible and highly porous framework, allowing for large strain under external pressure. BZT/BTO was introduced as a piezoelectric phase to provide energy generation under mechanical stimuli. Ppy and CNTs establish a percolated conductive network responsible for piezoresistive sensing. PDMS acts as a flexible and elastic matrix that encapsulates the structure, improving mechanical robustness, structural stability, and durability under repeated deformation. The material design allows the development of a dual-functionality sponge-based sensor system. Each component in this study was selected based on its well-established functionality and our previous experience rather than through an extensive multi-parameter optimization. PPy, CNTs, and piezoelectric phases were chosen to fulfill distinct and complementary roles, as detailed in the literature and in our earlier studies [26]. The advantages of each component are summarized in light of relevant references. Therefore, the focus of the present work is on demonstrating the feasibility and dual-function integration of the proposed material architecture.

As demonstrated above, while many researchers have developed pressure sensors using polyurethane sponges, a significant gap still exists in the development of polyurethane sponge pressure sensors capable of energy generation under mechanical stimuli through a piezoelectric mechanism. To our knowledge, no previous studies have reported a BZT- or BTO-coated polyurethane sponge pressure sensor with energy generation capabilities. Only a limited number of reports exist on sponge-based pressure sensors demonstrating energy generation performance [27]. Therefore, this work provides a significant contribution to the development of sponge pressure sensors with integrated energy generation under mechanical stimuli.

In this work, it is important to clarify that the proposed device is not designed as a hybrid sensing system, in which piezoresistive and piezoelectric effects simultaneously contribute to a single pressure-sensing signal. Instead, the two mechanisms coexist within the same structure while serving distinct and functionally decoupled roles. Pressure sensing is governed by the piezoresistive response of the conductive polypyrrole/carbon nanotube (PPy/CNT) network embedded in the polydimethylsiloxane (PDMS) matrix, resulting in a stable and continuous resistance-based signal under quasi-static compression. In contrast, the piezoelectric effect originating from the porous Ba(Zr,Ti)O_3_/BaTiO_3_ (BZT/BTO) phase is utilized for energy generation, generating a transient electrical output under dynamic mechanical excitation. Because of the differences in material phases, electrical output modes, and working conditions, the sensing and energy generation functions work in a functionally separate manner with negligible mutual interaction.

In this study, polyurethane sponge substrates were coated with a BZT/BTO film through a sol–gel process, while pyrrole was polymerized via an in situ polymerization approach. Subsequently, the coated substrates were exposed to a further coating process involving COOH-functionalized carbon nanotubes (f-CNTs) and PDMS. A comparative assessment of sensor performance was conducted between the samples coated with f-CNTs and those without this modification. It was expected that the hierarchically coated sponge substrate could confer energy generation capabilities and pressure-sensing capabilities under mechanical stimuli.

## 2. Experimental Section/Methods

### 2.1. Materials

Polyurethane sponge (with a mass of 74 g/m^2^, having a density of 14 deniers per square inch) was employed as the underlying substrate. The substrates underwent treatment with 1 mL/L of Triton X-100, a nonionic surfactant, in a Wascator washing machine (Electrolux Professional, Ljungby, Sweden) at a temperature of 60 °C, according to ISO 6330 2B guidelines [28]. Post-treatment, the substrates were subjected to drying in a tumbler dryer. Reagents employed in this study were of reagent-grade quality. These include phytic acid solution, ethanol, barium nitrate (Ba(NO_3_)_2_), zirconium(IV) acetylacetonate, titanium isopropoxide, sodium dodecyl benzene sulphonate (SDBS), cetyl trimethyl ammonium bromide (CTAB), pyrrole, ferric chloride hexahydrate (FeCl_3_·6H_2_O), hydrochloric acid (HCl), nitric acid (HNO_3_), sulfuric acid (H_2_SO_4_), sodium dodecyl sulfate (SDS), and polydimethylsiloxane silicon elastomer and curing agent (Sylgard 184 A and B), purchased from Aldrich^®^ (St. Louis, MO, USA), and multiwalled carbon nanotubes procured from Nanografi Co. (Ankara, Turkey). The conductive stainless yarns were provided by Adafruit Company (New York, NY, USA).

The composition and processing routes of the material system were determined based on previously established formulations and proof-of-concept considerations rather than systematic optimization.

PPy coating parameters were adopted from our previous studies on conductive textile coatings [29], where its processability and electrical performance were already validated. Therefore, PPy-related parameters were not re-optimized in the present work. Carboxylated CNTs were incorporated to introduce microstructural roughness and to support the formation of conductive pathways. The CNT layer was deposited using a dipping method on PU sponges. Optimization of CNT concentration, coating homogeneity, and processing parameters was beyond the scope of this study and is considered future work. BZT/BTO coating solutions were prepared and used as reference ceramic piezoelectric materials due to their well-documented piezoelectric performance. The selection of these materials reflects the continuity of our research trajectory, which includes our previous investigations on ZnO-based piezoelectric coatings [30] as well as our ongoing studies focused on PVDF nanofiber-based piezoelectric systems. It should be stated that an extensive optimization of material compositions and processing parameters is outside the scope of the present study and will be managed in future systematic investigations. The material selection was carried out with regard to previously established studies and functional considerations, with a focus on demonstrating a dual-functionality architecture rather than an optimized formulation.

### 2.2. BZT/BTO Coating by Sol–Gel Process

**Figure 1:**
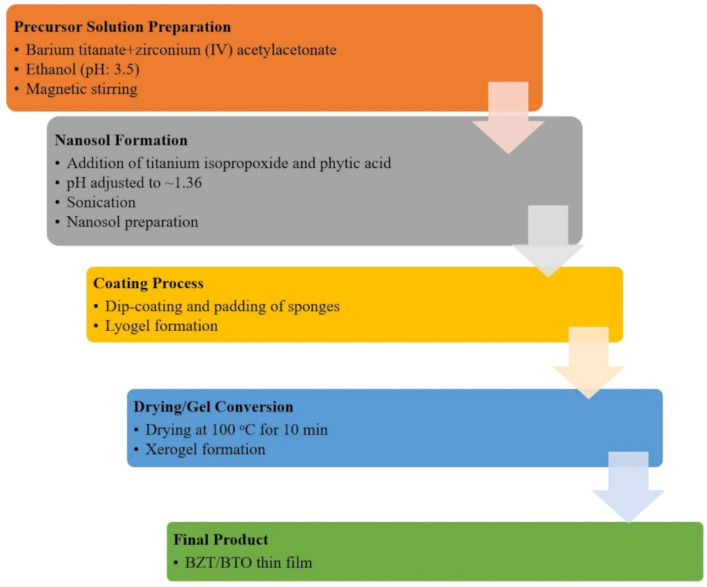
BZT/BTO thin film fabrication by sol–gel process on sponge samples.

An amount of 2.6 g of barium nitrate (Ba(NO_3_)_2_) and 2.43 g of zirconium(IV) acetylacetonate were separately dissolved in 150 mL of ethanol and subsequently mixed under magnetic stirring. Following this, the resultant solutions were mixed to adjust their pH to 3.5. Subsequently, 3 mL of titanium isopropoxide and 4 mL of phytic acid were introduced into the mixture, leading to a pH of 1.36, and the solution was sonicated for one hour. The sponge substrates were immersed in the solution, subjected to padding, and subsequently dried at 100 °C for 10 min. The flowchart of the process is illustrated in Figure 1. The coated sponge substrates were designated as “SBZT” for reference.

### 2.3. Polypyrrole Coating by In Situ Polymerization

A solution of 30 mL of pyrrole was prepared by dissolving it in 500 mL of distilled water. To this solution, 14.4 g of SDBS and 5 g of CTAB were introduced. The substrates were dipped in the pyrrole solution for a duration of 30 min, subsequently withdrawn, and subjected to rinsing with ethanol and distilled water. In a separate preparation, a solution was formed by dissolving 33.75 g of FeCl_3_·6H_2_O in 500 mL of distilled water. The treated substrates were immersed in this oxidative solution, allowing polymerization for 2 h. Following this stage, the treated fabric underwent immersion in a 1 M HCl solution (1 L) for a duration of 30 min, followed by rinsing and subsequent drying at a temperature of 50 °C as in our previous research [26,29]. The fabric samples coated with PPy were designated as “SBZTPPy” for future reference.

### 2.4. Carbon Nanotube Coating Process

At first, MWCNTs were activated in a process to introduce –COOH functional groups onto their surface. In this procedure, 2 g of MWCNTs was added to a mixture of 62.5 mL of HNO_3_ and 187.5 mL of H_2_SO_4_. The resulting mixture was subjected to sonication for two hours and allowed to rest overnight, followed by periodic rolling. Subsequently, the solution was gradually added dropwise into 500 mL of water, and the resultant mixture was decanted over a two-hour period. Afterward, the material was washed and centrifugated (TD3 centrifuge) at 4000 rpm for 10 cycles, followed by filtration. The final product was then dried at a temperature of 110 °C for a duration of 3 h [26,31]. In a subsequent step, 100 mg of the activated MWCNTs was dissolved in 100 mL of distilled water, and an additional 100 mg of sodium dodecyl sulfate (SDS) was introduced to the solution. This mixture was sonicated for 30 min. The substrates were immersed in this solution, subjected to padding, and subsequently dried at a temperature of 100 °C for a duration of 10 min. This pad–dry–cure process was carried out three times as in our previous work [26]. The substrates coated with MWCNTs were designated as “SBZTPPyCNT” for future reference.

### 2.5. Silicone Elastomer Film Production

**Figure 2:**
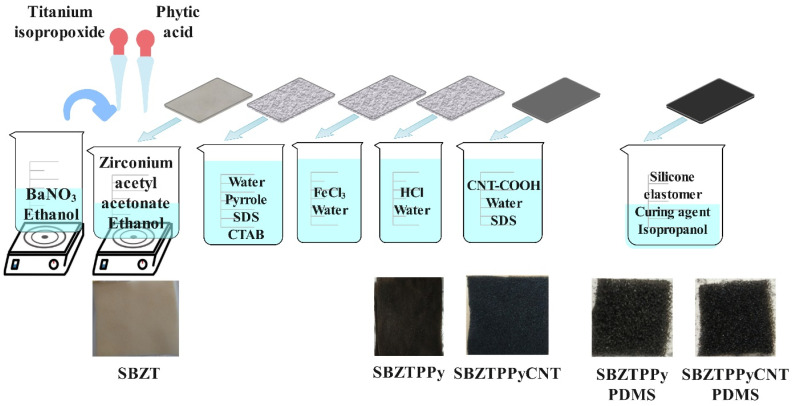
Schematic experimental process and digital images of coated samples.

The coating formulation was prepared by combining PDMS (Sylgard 184 A) (1.25 g) and curing agent (Sylgard 184 B) (0.12 g) within 50 mL of isopropanol, within an ultrasonic bath. Subsequent to immersion of fabrics into the solution, ultrasonic waves were employed for mixing for 15 min. The resultant specimens were air-dried overnight at ambient temperature and subsequently cured at 80 °C for a period of 1 h, following previously established protocols [26,32,33,34]. All experiments were performed three times. The PDMS-coated substrates were denoted as “SBZTPPyCNTPDMS” and “SBZTPPyPDMS” for reference. The schematic experimental process and photos of coated samples are shown in Figure 2.

### 2.6. Fabrication of Polyurethane Sponge Pressure Sensors

**Figure 3:**
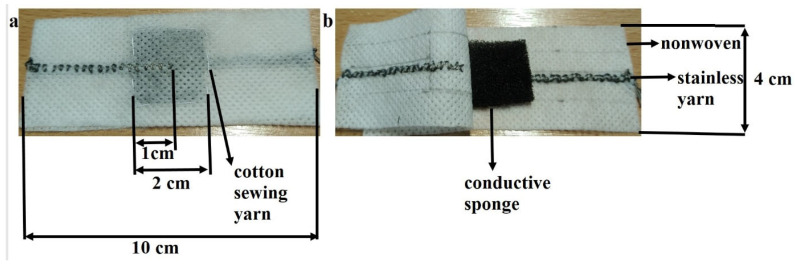
Digital images of flexible polyurethane sponge pressure sensor design: (**a**) sewing polyurethane sponge pressure sensor assembly with cotton yarn; (**b**) open polyurethane sponge pressure sensor assembly.

Wearable and flexible conductive sponge specimens were cut into square samples of 2 cm × 2 cm dimensions. The conductive sponges were enveloped within a polypropylene (PP) spunbond nonwoven fabric, onto which a stainless steel conductive yarn electrode was intricately embroidered. Hence, the study resulted in the design of fully textile-based wearable and flexible sensor assemblies, which offer notable advantages for wearable electronics. Stainless conductive yarns embroidered on PP nonwoven fabric were connected to a multimeter, an electrochemical workstation, and an oscilloscope for electrical and electromechanical analysis. The digital images of the flexible polyurethane sponge pressure sensor design are depicted in Figure 3.

### 2.7. Characterization of Conductive Polyurethane Sponges

Sample thickness measurements were conducted utilizing an electronic pressure transference apparatus developed at the University of Bolton (UK) [35,36]. The add-on values of the coated samples were ascertained through the application of the formula (W − W_o_)/W_o_, where W_o_ and W represent the weights of the uncoated and coated fabrics, respectively. The morphologies and structural characteristics of the specimens were examined employing scanning electron microscopy (SEM, Hitachi S-3400N, Tokyo, Japan). SEM analysis, conducted at an accelerating voltage of 20 kV, facilitated the depiction of surface microstructures of the coated samples. Prior to SEM imaging, a gold coating was applied to the sample surfaces. Additionally, energy-dispersive X-ray spectroscopy (EDS) was performed to investigate the elemental composition present on the surfaces of the coated specimens. For X-ray diffractometer (XRD) analysis of the SBZT samples, the APD 2000 PRO model device made by GNR (Novara, Italy) was used with Cu Kα radiation, operating at a scan rate of 1.2° per minute in the range of 10–80°, with a step size of 0.02° (2θ) and a dwell time of 1 s. Electrical resistance measurement of the conductive sponges was conducted at a 1 cm point distance via a digital multimeter (Keithley 2100 6 ½ Digital Multimeter, resistance range: 1 Ω–100 MΩ, Keithley Instruments, Cleveland, OH, USA).

### 2.8. Characterization of Polyurethane Sponge Pressure Sensors

The influence of diverse applied pressures on resistance, as well as response and recovery times, stability over multiple cycles, sensitivity, operational range, and detection limits, was assessed. The electromechanical performance evaluation of the sensor was executed using an Instron 3369 universal testing machine, Instron Corporation, Norwood, MA, USA. Simultaneously, the sensor’s corresponding resistance variation under diverse pressure conditions was directly measured employing a Keithley 2100 6 ½ Digital Multimeter. Current–voltage (I–V) characteristics of the pressure sensors were determined via two-electrode experiments and linear sweep voltammetry techniques, employing an electrochemical workstation (CHI 601D, CH Instrument Inc., Shanghai, China). Furthermore, real-time monitoring of various human movements, including finger, wrist, elbow, and knee bending, walking, jumping, and swallowing, was executed as shown in our previous research (Figure S1) [26]. Quasi-static compressive tests were performed using a universal tensile testing machine, where the samples were slowly compressed at a low loading rate to evaluate the piezoresistive pressure-sensing performance. Dynamic cyclic compressive loading was conducted using a linear motor at a frequency of 2 Hz to characterize the piezoelectric energy generation. The measurement of peak-to-peak output voltages of sensors exposed to forces via a linear motor was performed using Digital Real-Time oscilloscopes (TDS220, Tektronix Inc., Beaverton, OR, USA). To obtain loading force data, sensor samples were placed on the base of a reciprocating motor, and force measurements were gathered via a force sensor. For the purpose of systematic assessment, a linear motor test system (LinMot E1100-COXC, LinMot^®^/NTI AG, Spreitenbach, Switzerland) was employed (Figure S1). This system, controlled by computer software, facilitated the generation of periodic forces at a peak velocity of 0.157 m/s, a peak acceleration of 1.97 m/s^2^, and a frequency of 2 Hz, all under a force of 0.25 MPa [30]. The statistical assessment of the differences in peak-to-peak voltage values between SBZTPPyPDMS and SBZTPPyCNTPDMS samples was carried out via analysis of variance (ANOVA) using the SPSS software package (Version 20).

## 3. Results and Discussion

### 3.1. Conductive Polyurethane Sponges

#### 3.1.1. SEM-EDS Analysis

**Figure 4:**
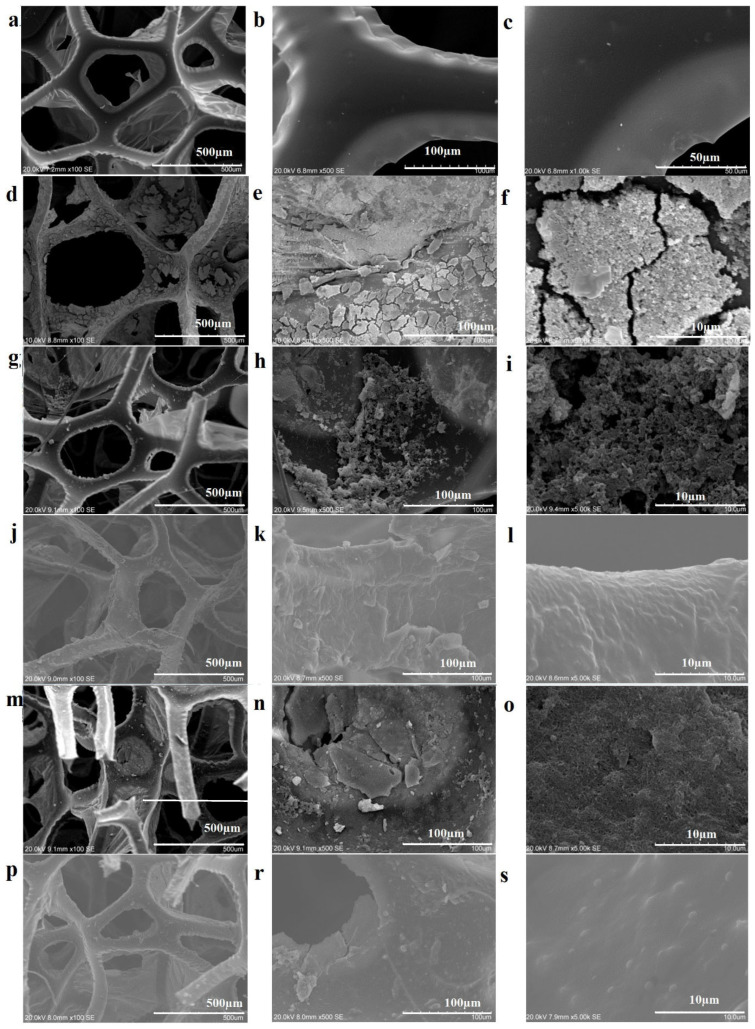
SEM images of (**a**–**c**) pristine sponge; (**d**–**f**) SBZT, (**g**–**i**) SBZTPPy, (**j**–**l**) SBZTPPyPDMS, (**m**–**o**) SBZTPPyCNT, and (**p**–**s**) SBZTPPyCNTPDMS fabric samples.

The PU sponge is characterized by its typical porous and three-dimensional structure, with homogeneous and smoothly shaped polygonal cells with a frame width of about 50 µm, a pore diameter of 200–500 µm, and a uniform pore framework (Figure 4a–c). After dip coating and drying, the sponges preserve their initial structural integrity while simultaneously exhibiting a notable enhancement in surface roughness throughout the scaffold. In particular, in SBZT and SBZTPPy samples, attachments were observed on the cell edges, coexisting with the formation of wrinkles in the film. In SBZT samples, spherical protrusions and cracks are clearly visible on the rigid film (Figure 4d–i). SBZT samples with the physically adsorbed BZT/BTO thin film also exhibit a denser film layer between cell edges compared to SBZTPPy samples (Figure 4g–i). Globular PPy aggregates were evenly dispersed throughout the pore structure of the sponge. Polymerization of pyrrole promotes the development of a micro-nano hierarchical roughness on the surface. The uniform distribution of globular particles indicates the successful loading of PPy nanoparticles onto the surface. Subsequent CNT coating, following PPy deposition, results in the filling of interstitial spaces between PPy aggregates (Figure 4m–o). Specifically, the PPy particles are present in an aggregated state with a spherical morphology. After the completion of PPy deposition and CNT and PDMS coating, the sponge’s porous structure remains intact and the coatings do not occlude the sponge’s pores. The PDMS coating covers the aggregated frame surfaces, securely adhering to particles, nanotubes, and PPy aggregates (as shown in Figure 4j–l,p–s). This observation confirms that the coatings effectively preserve the open-pore structure of the sponge while providing surface coverage to the frames and edges [37,38].

**Figure 5:**
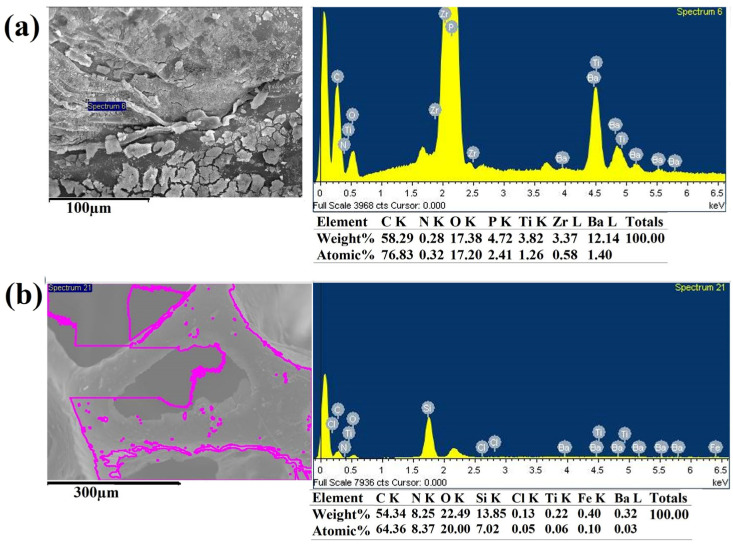
EDS spectra of the sensor samples (**a**) SBZT and (**b**) SBZTPPyCNTPDMS.

EDS analysis validated the presence of zirconium, barium, titanium, phosphorus, carbon, and oxygen in the SBZT samples. After PPy coating, the presence of nitrogen, iron, and chloride was confirmed, while silicium content was detected after PDMS coating (as summarized in Table 1 and shown in Figure 5 and Figure S2). It is worth noting that phosphorus and zirconium elements were no longer detectable after PDMS coating. This observation highlights the efficacy of EDS as a robust technique for characterizing the surface of composites, with a specific emphasis on their suitability for surface analysis as opposed to bulk characterization [39,40,41].

The sensing performance of the sponge-based sensor is closely related to its hierarchical microstructure, as revealed through SEM and EDS analyses. The pristine PU sponge, with a typical three-dimensional porous architecture and uniformly distributed polygonal cells, provides high compressibility and efficient stress transfer under external loading. The rigid porous BZT/BTO layer generates spherical protrusions and microcracks on the cell edges, creating localized stress concentration sites that improve piezoelectric polarization under dynamic compression. Globular PPy aggregates formed throughout the pore walls, causing the introduction of a micro–nano hierarchical roughness and thus increasing the number of conductive junctions. Subsequent CNT deposition fills the interstitial spaces between PPy aggregates, establishing a percolated and deformation-sensitive conductive network responsible for the piezoresistive pressure-sensing behavior. Significantly, the final PDMS coating encapsulates the PPy/CNT network without blocking the pores, ensuring elastic recovery, mechanical stability, and long-term cycling durability. This well-preserved open-pore architecture enables stable resistance modulation under quasi-static compression while maintaining efficient piezoelectric energy generation under dynamic excitation, thereby demonstrating a clear structure–function relationship.

#### 3.1.2. XRD Analysis

**Figure 6:**
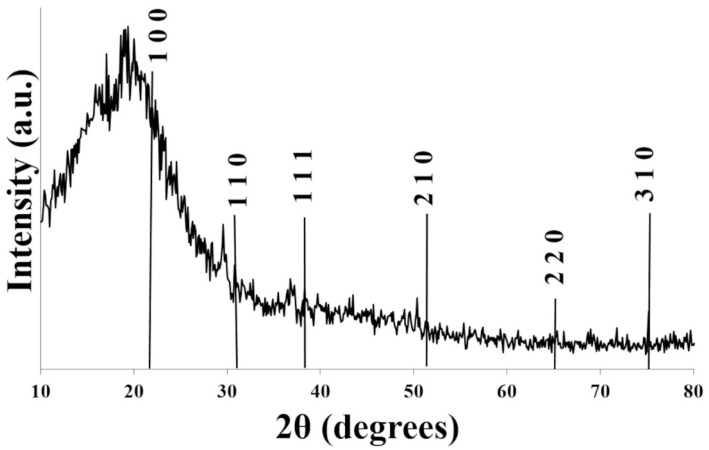
XRD pattern of SBZT sample.

The XRD pattern of the SBZT sample is shown in Figure 6. The diffractogram shows weak signals at 22.2, 31.5, 38.9, and 51.1, which could be assigned to (100), (110), (111), and (210) crystallographic planes of tetragonal perovskite BaTiO_3_ (ABO_3_). The peaks of Ba(Zr,Ti)O_3_ are similar to BTO, but are slightly shifted due to Zr. The BZT peaks overlapped with BTO peaks and were weak due to poor crystallinity. The weak peaks near 25° and 27–28° could be attributed to TiO_2_ and ZrO_2_/BaO as possible secondary phases, respectively. Polyurethane sponge has an amorphous nature, which is attributed to a broad amorphous peak around 18–22° (2θ). The XRD pattern shows the amorphous PU matrix and dispersed crystalline BZT + BTO, along with possible minor secondary phases [42]. It should be noted that the diffraction peaks associated with the BZT/BTO phase exhibit relatively low intensity. This behavior is primarily attributed to the low loading of the ceramic phase within the composite and its porous microstructure, which reduces the effective coherent scattering volume. Similar reductions in peak intensity have been reported for flexible polymer–ceramic composites designed for wearable sensing applications [43].

#### 3.1.3. Electrical Properties

Table 2 lists the add-on, thickness, and resistance values of the specimens. Following the coating procedures, the add-on values exhibited a gradual increment, whereas the thickness values demonstrated negligible alteration. Electrical characteristics change when sponges are coated with conductive polymers. After the application of PPy coating, the resistance values decreased significantly, falling within the kilohm range. In contrast, the resistance of the pristine sponge exceeded the measurement limit of the multimeter, set at a maximum of 100 MΩ. However, the introduction of CNT coating resulted in a slight elevation in resistance, caused by Cl^−^ ion removal by the dedoping of PPy, as confirmed by EDS analysis (Table 1). Furthermore, the application of dielectric PDMS coating led to a further rise in resistance, although values remained within the kilohm range.

### 3.2. Polyurethane Sponge Pressure Sensors

#### 3.2.1. Sensing and Energy Generation Mechanisms

**Figure 7:**
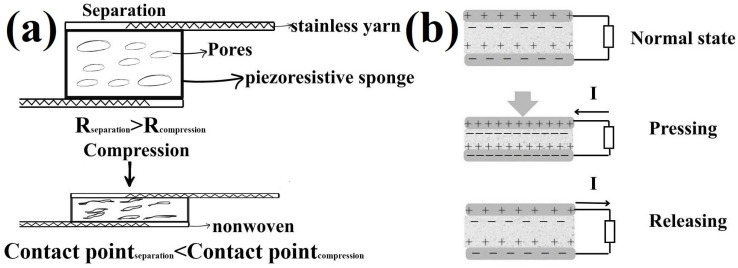
The piezoresistive (**a**) and piezoelectric (**b**) working mechanism of the polyurethane sponge sensor assemblies.

The working mechanism of the produced polyurethane sponge-based sensor assembly is governed by its piezoresistivity together with piezoelectricity. Conductive material coatings such as PPy and CNTs contribute to the piezoresistive effect by means of the contact–separation mechanism, enhancing conductive pathways within the porous sponge structure. The resistance of sponge-based sensor assemblies decreases due to the increased contact area and the formation of additional conductive pathways within the sponge pores, as well as between the sponge and the embroidered stainless yarns under compression. Once external compression is applied, the number of contact points and contact planes increases across the conductive PPy layer, BZT/BTO, and CNT coating on the sponge while the pores collapse. After the compression is released, the flexible sponge returns to its normal state, restoring its initial resistance with excellent cyclic stability due to the inherent elasticity of the polyurethane sponge (Figure 7a) [26].

Energy harvesting was demonstrated in terms of energy generation capability under mechanical stimuli rather than as an independent nanogenerator system. Alongside the piezoresistive sensing function, the material exhibits the ability to generate electrical output under mechanical excitation, confirming the coexistence of sensing and energy generation functionalities within a single dual-functional structure. The piezoelectric working mechanism of a terry towel coated with ZnO nanoplates as piezoelectric material, PPy, and CNTs has been discussed in our previous research [30]. Here, the piezoelectric contribution of a BZT/BTO thin film structure for polyurethane sponge sensors is highlighted. The high surface area, charge density, dielectric properties, and rough surface structure created by the BZT/BTO thin film could lead to energy generation capability under mechanical stimuli in polyurethane sponge sensors [11]. In a normal state, no external force is applied to the piezoelectric nanogenerator. Positive and negative charges are distributed on the top and bottom electrodes in a balanced manner. The piezoelectric BZT/BTO@PPy/CNT/PDMS-coated polyurethane sponge possesses intrinsic dipole moments in the crystal structure of BZT/BTO; however, net charge transfer does not occur to the outside. There is no electron flow (I). In the compression phase, an external compression is applied to the nanogenerator assembly. Under the applied compression, the piezoelectric material undergoes mechanical deformation. The orientation of dipole moments in the crystal lattice changes, and the charges accumulate at the top and bottom of the piezoelectric layer. This charge accumulation creates a potential difference (piezoelectric voltage) between the electrodes, which drives electrons to flow between electrodes, generating a current (I). When the compression is released, the sponge recovers to its normal state, generating a potential difference in the opposite direction. Consequently, the current (I) flows in the reverse direction compared to the compression state. Therefore, each compression–release cycle produces electrical energy in an alternating current (AC) form. Every compression–release cycle corresponds to one period of the piezoelectric output signal (Figure 7b). The piezoelectric effect of BZT/BTO materials has been reported in previous studies [44,45]. The composite coating on sponges with BaTiO_3_, PPy, and CNTs, which creates a rough surface with enhanced charge density and permittivity, could exhibit high sensor output performance together with energy generation properties under mechanical stimuli [46].

Although both piezoresistive and piezoelectric components are integrated within the same device architecture, their functional roles are clearly separated. Under quasi-static compressive loading applied by the tensile tester, the sensor exhibited stable and continuous resistance variation, indicating that the pressure-sensing behavior is governed by the piezoresistive response of the PPy/CNT conductive network. In contrast, under dynamic cyclic compression at 2 Hz applied by the linear motor, an alternating voltage output was generated due to the stress-induced polarization of the porous BZT/BTO phase. Importantly, the resistance-based sensing signal showed no measurable interference during dynamic excitation, confirming that the piezoresistive sensing and piezoelectric energy generation functions operate in a decoupled manner.

#### 3.2.2. Electromechanical Analysis

**Figure 8:**
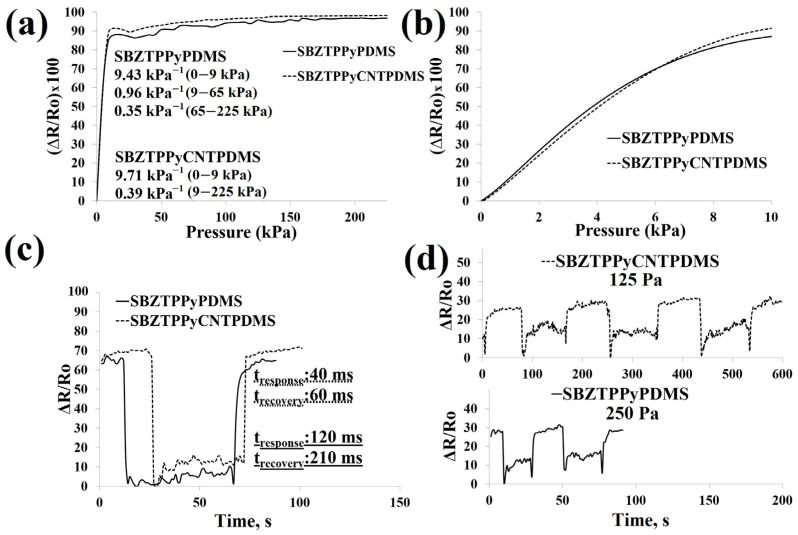
(**a**) Sensitivity graph between 0 and 225 kPa, (**b**) sensitivity graph between 0 and 10 kPa, (**c**) response and recovery time (12.5 Kpa), and (**d**) detection limit for SBZTPPyPDMS and SBZTPPyCNTPDMS samples.

**Figure 9:**
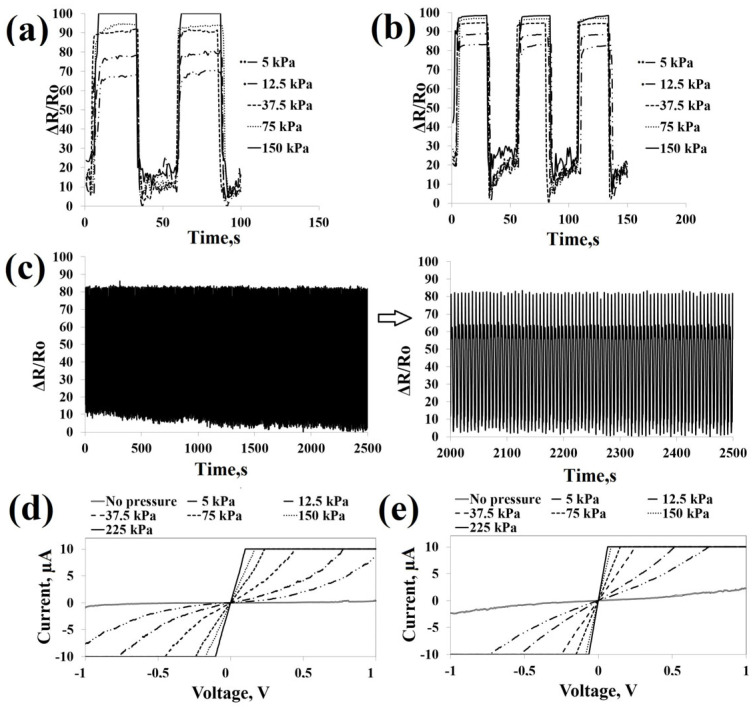
(**a**,**b**) Resistance change under various pressures on SBZTPPyPDMS and SBZTPPyCNTPDMS, (**c**) durability under multiple loading–unloading cycles (37.5 kPa) of SBZTPPyCNTPDMS, and (**d**,**e**) current–voltage (I–V) curves under various pressures on SBZTPPyPDMS and SBZTPPyCNTPDMS, respectively.

Table 3 presents the sensitivity, detection limit, response time, recovery time, durability, and peak-to-peak voltage values of the samples. The impact of different applied pressures on resistance, along with the response and recovery times (12.5 kPa), detection limit, stability during multiple cycles, sensitivity in the range of 0–225 kPa, and current–voltage (I–V) curves, is illustrated in Figure 8 and Figure 9. The porous three-dimensional architecture provides the capability for variable stress sensing, which is advantageous for fabricating motion-detection sensors with enhanced wearer comfort [47]. The resistance of the sponges depends upon their voluminous, three-dimensional, and porous composition, which is influenced by compression. The presence of PPy aggregates enveloping the sponge surface is favorable for enhancing electron transfer efficiency and enabling spatial deformation. Importantly, contact points between conductive pathways offer opportunities for the formation of new interconnections, particularly under applied pressure. SBZTPPyPDMS and SBZTPPyCNTPDMS sensor assemblies exhibited a similar sensitivity level of approximately 9.5 kPa^−1^ within the lower-pressure range spanning from 0 to 9 kPa. However, within the higher-pressure range of 9 to 225 kPa, SBZTPPyPDMS displayed a slightly elevated sensitivity of 0.96 kPa^−1^ compared to SBZTPPyCNTPDMS (as illustrated in Figure 8a,b, and detailed in Table 3). Furthermore, SBZTPPyCNTPDMS demonstrated a rapid response (40 ms) and recovery time (60 ms), and a lower detection threshold of 125 Pa in contrast to SBZTPPyPDMS (as shown in Figure 8c,d and summarized in Table 3). The results show a significant improvement in the performance of the pressure sensor due to the presence of CNT coating. Furthermore, the resistance variation (ΔR/R_o_) reached 68% for SBZTPPyPDMS and 82% for SBZTPPyCNTPDMS, both at an applied pressure of 5 kPa. At a higher applied pressure of 150 kPa, the resistance change reached 100% for both sensors (Figure 9a,b). SBZTPPyCNTPDMS exhibited superior stability across more than 1125 cycles compared to SBZTPPyPDMS over >750 cycles (Figure 9c). In this study, the primary objective is to evaluate the stability and durability of the electrical responses under cyclic quasi-static compressive loading rather than to investigate detailed mechanical deformation. The results demonstrate that both electrical responses remain stable over repeated loading–unloading cycles, indicating robust mechanical integrity and reliable functional performance of the material system under cyclic deformation.

Furthermore, as the applied force increased from 5 kPa to 225 kPa, the slope of the current–voltage (I–V) curve exhibited an upward trend (Figure 9d,e), signifying adherence to Ohm’s law by the sensors. In Table 4, the results of variance analysis for the peak-to-peak voltage values of SBZTPPyPDMS and SBZTPPyCNTPDMS are presented. The variance analysis indicated a significant distinction between the peak-to-peak voltage values of SBZTPPyPDMS and SBZTPPyCNTPDMS. Notably, SBZTPPyCNTPDMS exhibited a peak-to-peak voltage value of 1.25 V, output power of 0.93 µW, and power density of 2.31 mW/m^2^, indicating its energy generation capability under mechanical stimuli. The power generation capacity of the sensor might be attributed to the presence of the BZT/BTO film on the sensors. Furthermore, according to Table S1, there are no reported studies that integrate piezoelectric and piezoresistive performance in sponge-based pressure sensors.

Although the output voltage and power density obtained in this study are lower than those reported for recently developed hybrid nanogenerators, the primary aim of the present work is not to maximize nanogenerator performance. Instead, the results demonstrate the feasibility of mechanical energy generation in conjunction with piezoresistive pressure sensing within a single dual-functional sensor. As highlighted in Table S1, studies that concurrently address both piezoresistive sensing and energy generation remain scarce. Therefore, the integration of these functionalities within a unified sponge-based architecture constitutes a key innovation of this work. Further efforts will be directed toward enhancing energy generation efficiency through compositional optimization, microstructural engineering, and improved electrical interface design.

#### 3.2.3. Real-Time Human Motion Monitoring

To demonstrate practical applicability, the sensor was attached to different body parts and evaluated under real-time human activities, including finger, wrist, elbow, and knee bending, as well as walking, jumping, and swallowing. Each motion produced a distinct and stable pressure signal, reflecting differences in deformation amplitude and frequency. These results indicate that the proposed sensor is capable of monitoring both large-scale body movements and subtle physiological motions, highlighting its potential applications in wearable electronics and real-time human motion analysis.

**Figure 10:**
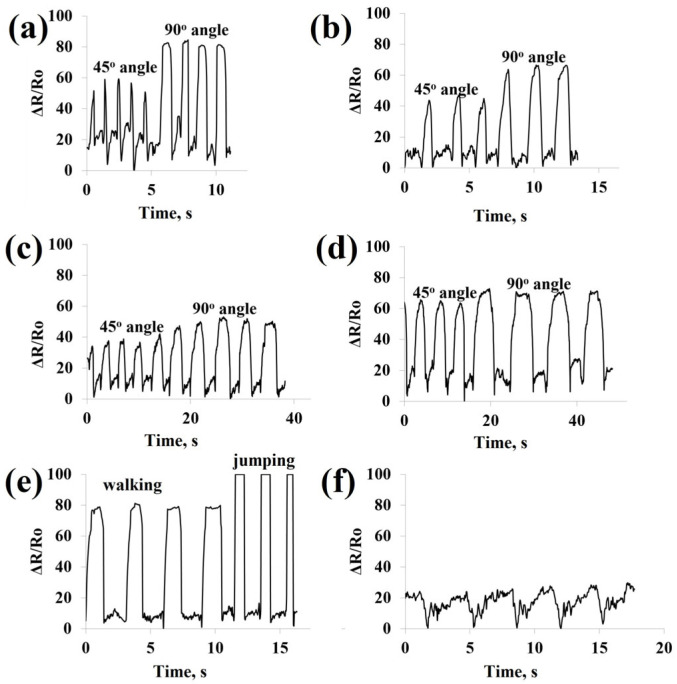
Resistance changes for SBZTPPyCNTPDMS samples under various human motions such as (**a**) finger bending of 45° and 90°, (**b**) wrist bending of 45° and 90°, (**c**) elbow bending of 45° and 90°, (**d**) knee bending of 45° and 90°, (**e**) walking and jumping, and (**f**) swallowing in real time.

Figure 10 displays resistance variation graphs for SBZTPPyCNTPDMS samples in response to various human motions, including finger, wrist, elbow, and knee bending, walking, jumping, and physiological signals such as swallowing, captured in real time. The human movements are schematically illustrated in Figure S3. The outcomes highlighted the potential applicability of the sensor for human motion monitoring in health and sports. The sensor possesses the capability to detect a broad spectrum of motion. The sensor was affixed to the joints of the finger, wrist, elbow, knee, feet, and neck using tape. During bending of the finger, wrist, elbow, and knee, walking, jumping, and swallowing, the sponge-based sensor exhibited resistance variation because of pronounced compression.

The various movements and diverse angles can be clearly distinguished through a comparative analysis of resistance variation. These findings confirm excellent sensing capability and repeatability of the sensor for human motion detection. Beyond its ability to sense a wide range of motions, the sponge-based pressure sensor is also capable of detecting subtle pressures. To assess the effectiveness of the sensor in capturing tiny human activities, it was affixed to the neck to non-invasively monitor muscular movements near the throat during swallowing. In real time, the sensor recorded resistance fluctuations associated with the swallowing (Figure 10f). This demonstrates the sensor’s ability for diagnosing subtle physiological signals and detecting a broad range of movements, emphasizing its potential applications in both medical diagnostics and sports performance assessment.

## 4. Conclusions

This study proposes that the BZT/BTO@PPy/CNT/PDMS-coated polyurethane sponge sensor (SBZTPPyCNTPDMS) can produce piezoresistive signals while harvesting mechanical energy via the piezoelectric effect, thus enabling highly sensitive wearable pressure sensing with built-in energy generation capability. In this work, the pressure-sensing and energy generation properties of a novel sensor design based on the SBZTPPyCNTPDMS assemblies were comprehensively explored. The study included XRD, SEM-EDS analysis, electromechanical and energy generation performance characterizations under mechanical stimuli, and evaluation of pressure-sensing performance across various human activities. The results yielded the following observations:

○ EDS analysis validated the presence of Ba, Zr, and Ti in the sponge samples coated via the sol–gel process, while the subsequent PPy coating introduced nitrogen, iron and chlorine presence, and PDMS application resulted in Si content on the sensor samples.
○ The presence of a rough surface structure in the coated samples without occluding the pores inherent to sponges was shown in the SEM analysis.
○ In particular, the sensor displayed exceptional pressure-sensing capabilities under quasi-static compressive loading across a wide range of pressures, showing a high sensitivity (approximately 9.5 kPa^−1^ within the pressure range of 0–9 kPa), a low detection threshold (125 Pa), remarkable cyclic stability (>1125 cycles), and rapid response (40 ms) and recovery time (60 ms). Furthermore, the incorporation of CNT coating caused an enhancement in sensor performance.
○ The sensor’s capability to monitor common human activities in real time was confirmed, emphasizing its potential for practical applications.
○ Under periodic dynamic loading–unloading cycles at 2 Hz, the sensor produced a peak-to-peak voltage of 1.25 V, an output power of 0.93 µW, and a power density of 2.31 mW/m^2^, confirming energy generation under mechanical stimuli.

Consequently, the prepared SBZTPPyCNTPDMS assemblies present an innovative approach for developing eco-friendly, cost-effective, and highly sensitive sponge pressure sensors, combining piezoresistive sensing with energy generation capability under mechanical stimuli. This dual functionality offers promising potential for wearable and flexible sensor applications. Future research on sponge-based piezoresistive sensors with dual functionality may concentrate on enhancing the energy generation efficiency under mechanical stimuli and the long-term mechanical robustness under repeated quasi-static deformation by employing multiple sponge layers. Tuning the microstructure, porosity, and surface characteristics of the sponge substrate could enable higher electrical output while preserving sensing accuracy. Furthermore, the development of fully textile-based structures will be a key step toward realizing practical wearable applications with enhanced comfort.

## Figures and Tables

**Table 1:** Elemental analysis of coated sponge samples.

Weight %/Standard Deviation	C	O	P	N	Ti	Zr	Ba	Si	Fe	Cl
**S**	58.04/6.09	24.99/2.88	-	16.98/7.68	-	-	-	-	-	-
**SBZT**	50.02/6.22	32.19/7.24	5.34/1.8	0.10/0.21	3.50/0.86	3.90/1.23	4.94/3.56	-	-	-
**SBZTPPy**	59.50/4.87	26.59/6.51	-	11.95/7.85	0.40/0.51	0.09/0.21	0.91/1.19	-	0.35/0.47	0.20/0.20
**SBZTPPyPDMS**	48.34/4.26	27.89/1.70	-	11.00/6.80	0.10/0.13	-	0.42/0.47	11.43/4.13	0.68/1.03	0.14/0.12
**SBZTPPyCNTPDMS**	47.25/7.41	17.76/8.58	-	7.87/2.86	0.14/0.13	-	1.29/1.82	24.96/13.6	0.33/0.51	0.41/0.36

**Table 2:** Add-on, thickness, and electrical resistance values of sponge samples.

	Add-On, % (SD *)	Resistance, kOhm (SD)	CV, %	Thickness, mm
**S**	-	-		4.50
**SBZT**	14.13 (1.74)	-		4.50
**SBZTPPy**	18.83 (1.02)	27 (6.3)	23	4.50
**SBZTPPyPDMS**	41.36 (2.40)	409 (91)	22	4.50
**SBZTPPyCNT**	29.93 (2.03)	74 (22)	30	4.50
**SBZTPPyCNTPDMS**	61.73 (3.11)	342 (154)	45	4.60

* SD: standard deviation, CV: coefficient of variation.

**Table 3:** The sensitivity, detection limit, response time and recovery time, durability, and peak-to-peak voltage values of samples.

	Sensitivity (kPa^−1^)	Detection Limit	Response Time, ms	Recovery Time, ms	Durability, Cycles	Peak-to-Peak Voltage
			(12.5 kPa)	(12.5 kPa)		Mean–SD
**SBZTPPyPDMS**	9.43 (0–9 kPa) 0.96 (9–65 kPa) 0.35 (65–225 kPa)	250 Pa	120	210	>750	1.05 V–0.16
**SBZTPPyCNTPDMS**	9.71 (0–9 kPa) 0.39 (9–225 kPa)	125 Pa	40	60	>1125	1.25 V–0.21

**Table 4:** One-way ANOVA analysis of peak-to-peak voltage values of SBZTPPyPDMS and SBZTPPyCNTPDMS.

	Sum of Squares	df	Mean Square	F	Sig.
**Between Groups**	1.00	1	1.00	28.07	0.000
**Within Groups**	3.45	97	0.04		
**Total**	4.45	98			

## Data Availability

The original contributions presented in this study are included in the article/Supplementary Material. Further inquiries can be directed to the corresponding author.

## Supplementary Materials

The following supporting information can be downloaded at https://www.mdpi.com/article/10.3390/polym18020241/s1. Figure S1. Electromechanical test equipments set-up Figure S2. EDS spectra of the sensor samples (a) SBZTPPy, (b) SBZTPPyPDMS. Figure S3. Schematically illustration of real-time human movements; (a) finger bending, (b) wrist bending, (c) elbow bending, (d) knee bending, (e) walking, (f) swallowing. Table S1. Some results in literature about polyurethane sponge pressure sensor coated conductive and piezoelectric materials. References [3,11,12,14,16,20,27,48,49,50,51,52] are cited in Supplementary Materials.

## Abbreviations

PDMS	Polydimethylsiloxane
PPy	Polypyrrole
PANI	Polyaniline
CNTs	Carbon nanotubes
MWCNTs	Multiwalled carbon nanotubes
rGO	Reduced graphene oxide
PU	Polyurethane
CB	Carbon black
HGM	Hollow glass nanosphere
ADP	Ammonium dihydroge phosphate
PTFE	Polytetrafluoro ethylene
FEP	Fluorinated ethylene propylene
TPU	Thermoplastic polyurethane
PEDOT:PSS	Poly(3,4-ethylenedioxythiophene) polystyrene sulfonate
V_OC_	Open-circuit voltage
I_SC_	Short-circuit current
GF	Gauge factor
A-CNT	Aminated carbon nanotube
LED	Light-emitting diode
Q_tr_	Transfer charge
BTO	Barium titanate, BaTiO_3_
BZT	Barium zirconium titanate, Ba(Zr,Ti)O_3_
PVDF	Polyvinylidene fluoride
SD	Standard deviation
SDS	Sodium dodecyl sulfate
SDBS	Sodium dodecyl benzene sulphonate
CTAB	Cetyl trimethyl ammonium bromide
PP	Polypropylene
SEM	Scanning electron microscopy
EDS	Energy-dispersive X-ray spectroscopy
FTIR	Fourier-Transform Infra-red Spectroscopy
XRD	X-ray diffractometer
ANOVA	Analysis of variance
