# Supplementary material for: Dual-Functional Polyurethane Sponge-Based Pressure Sensors Incorporating BZT/BTO, Polypyrrole, and Carbon Nanotubes with Energy Generation Capability
Source: Polymers (Basel). 2026 Jan 16;18(2):241. doi: 10.3390/polym18020241 (PMC12845847; doi:10.3390/polym18020241)
Supplement: Supplementary file 1 [file polymers-18-00241-s001.zip › polymers-4066800-supplementary.pdf]

Dual-functional Polyurethane Sponge-Based Pressure Sensors Incorporating BZT/BTO, Polypyrrole, and Carbon Nanotubes with Energy Generation Capability

Nurhan O. Camlibel, Baljinder K. Kandola*

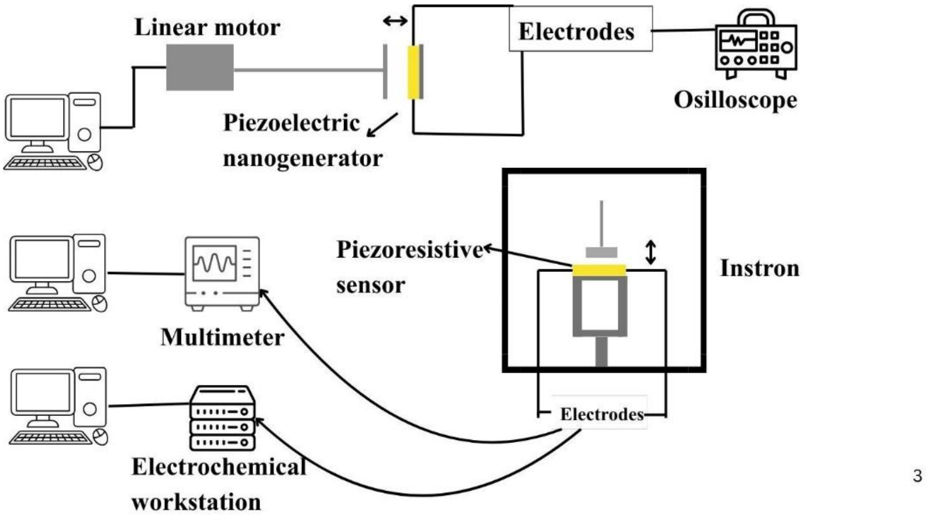

Figure S1. Electromechanical test equipments set-up

a)

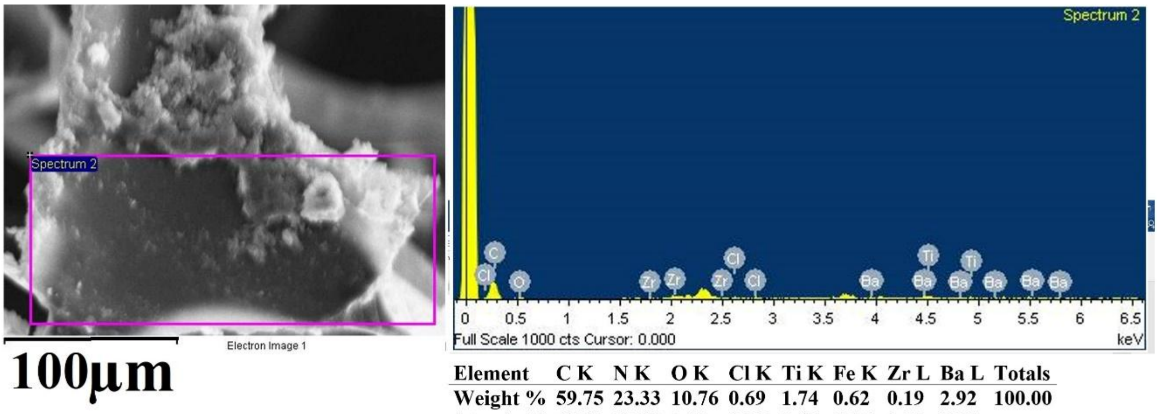

Element	C K	N K	O K	Cl K	Ti K	Fe K	Zr L	Ba L	Totals
Weight %	59.75	23.33	10.76	0.69	1.74	0.62	0.19	2.92	100.00
Atomic %	67.20	22.50	9.08	0.26	0.49	0.15	0.03	0.29	

b)

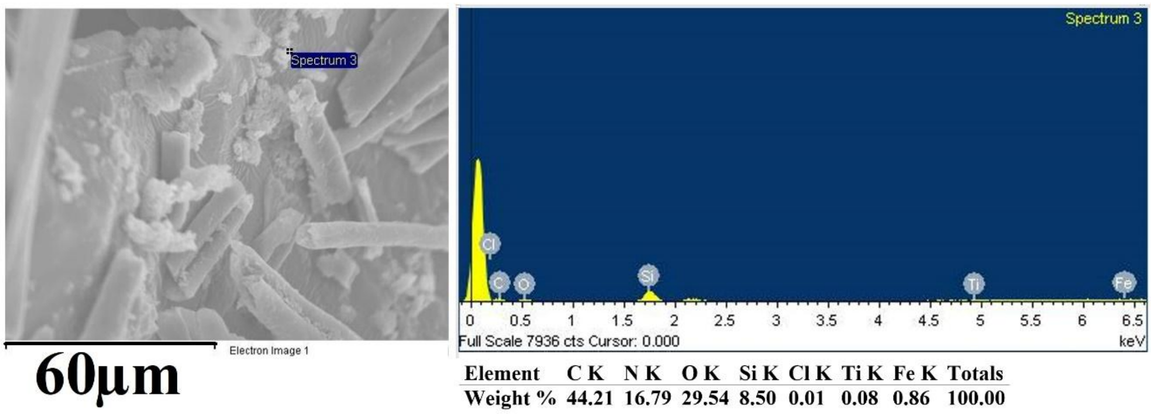

Element	C K	N K	O K	Si K	Cl K	Ti K	Fe K	Totals
Weight %	44.21	16.79	29.54	8.50	0.01	0.08	0.86	100.00
Atomic %	52.24	17.01	26.21	4.29	0.01	0.02	0.22	

Figure S2. EDS spectra of the sensor samples a) SBZTPPy, b)SBZTPPyPDMS.

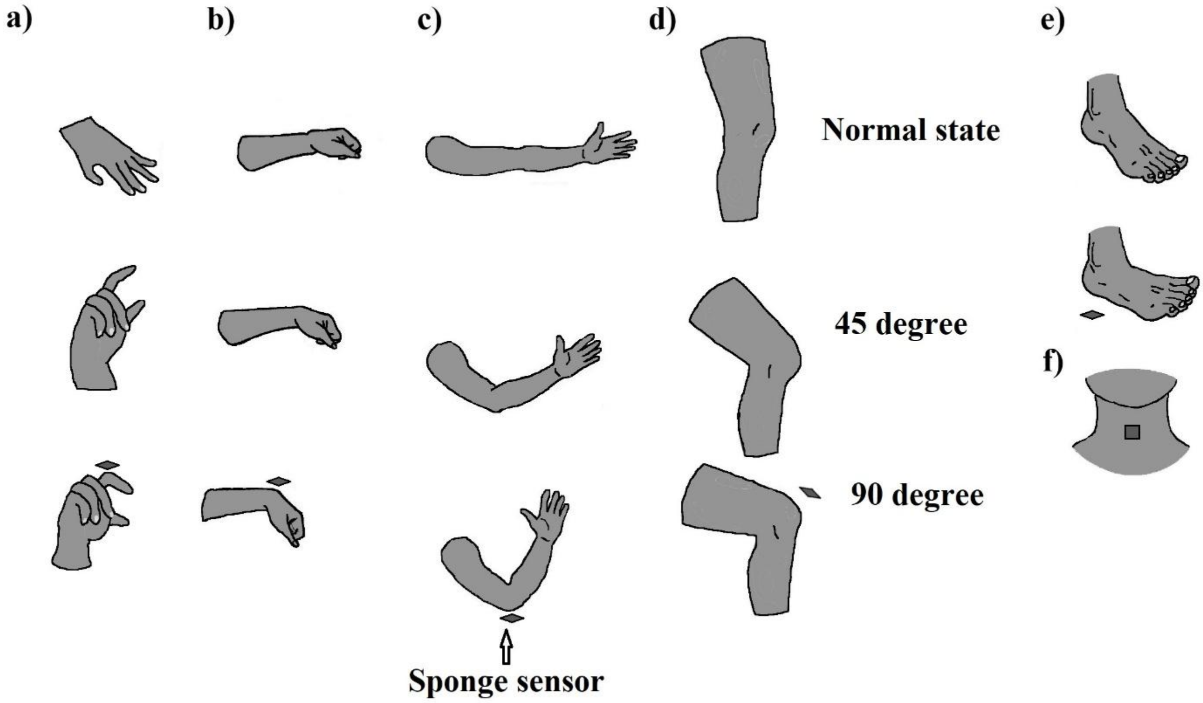

Figure S3. Schematically illustration of real-time human movements; a) finger bending, b) wrist bending, c) elbow bending, d) knee bending, e) walking , f) swallowing

Table S1. Some results in literature about polyurethane sponge pressure sensor coated conductive and piezoelectric materials

Mechanism	Coating materials	V_{OC}	I_{SC}	Sensitivity	Response time, recovery time	Working range	Repeatability, cycles	Application	Ref.
Piezoresistive<br>Piezoelectric	Zr-doped $BaTiO_3$/PPy/CNT/PDMS	1.25 V<br>Output power: 0.93 µW<br>Power density: 2.31 mW/m^2	-	9.71 (0-9 kPa)<br>0.39 (9-225 kPa)<br>Detection limit: 125 Pa	40/60 ms	0-225 kPa	>1125	Pressure sensor<br>Nanogenerator	This work
Piezoresistive	$BaTiO_3$/rGO	-	-	2.64 kPa^{-1}	560 ms	0-60 kPa	-	Pressure sensor	[3]
Piezoresistive	CB/CNT/TPU	-	-	0.1 kPa^{-1} (0–8 kPa)<br>0.01 kPa−1 (8–23.3 kPa)	119/59 ms	-	>1000	Pressure sensor	[12]
Piezoresistive	GO/PPy	-	-	0.79 kPa^{-1}	70 ms	75 Pa-15 kPa	>10000	Pressure sensor	[14]
Piezoresistive	CNT	-	-	2.7% kPa^{-1} (0-15 kPa)	60/100 ms	-	18000	Pressure sensor	[16]
Piezoresistive	Mxene/CNT	-	-	GF = 0.56 (0–19%), 1.19 (19–60% for stretching mode<br>GF= 3.55 (0–6%), −4.43(6–17%), −0.58 (17–66%), −2.30 (66–80%)<br>for compressive mode	206/439 ms (stretching strain)<br>583/407 ms (compressive strain)	-80% compressive strain,<br>60% stretching strain	5000	Pressure sensor	[48]
Piezoresistive	Graphene/AgNWs	-	-	GF: 2.39 (>51.18%)<br>GF: 0.66 (29.31-51.18%)<br>GF: 0.47 (<29.31%)	110/106 ms	70% compressive strain (62.75-31.79 kPa)	3000	Pressure sensor	[49]
Piezoresistive	A-CNT/HGM/ADP/PDMS	-	-	60.2 kPa^{-1} (0−1.45 kPa)<br>1.10 kPa^{-1} (1.45−7.1 kPa)<br>0.06 kPa^{-1} (7.1−45 kPa)	152/178 ms	0-45 kPa	>3000	Pressure sensor	[50]
Piezoelectric	ZnO/RGO	0.5 V	2 $\mu A/cm^2$	-	-	-	3000	Nanogenerator	[20]
Triboelectric	MWCNTs/PPy<br>Negative layer: PTFE membrane	110V, 235 LED lightening	12µA, Transfer charge: 30-46 nC	10-70% compression deformation	-	-	-	Nanogenerator<br>Pressure sensor	[11]
Triboelectric	CNT/Fe_3O_4<br>Negative layer: FEP film	V_{oc}:34.8 V<br>Output power: 147.9µW<br>Power density: 1.3$\mu W/cm^2$,<br>35 LEDs lightening	Q_{tr}:10.1 nC<br>I_{sc}: 267.1 nA	-	-	-	-	Nanogenerator	[51]
Piezoresistive<br>Triboelectric	CNT<br>Negative layer: PDMS film	252 V<br>Output power density: 1.642 W/m^2 at 10 MΩ<br>738 paralel LEDs lightening	38µA	8.23 kPa^{-1} (20 Pa − 20 kPa)<br>3.71 kPa^{-1}(20 kPa − 100 kPa)<br>1.65 kPa^{-1} (>100 kPa)	88/90 ms	-80% compressible, 20 Pa-100 kPa	8000	Pressure sensor<br>Nanogenerator	[27]
Triboelectric	PANI<br>Negative layer: PTFE ball	540 V	6 µA	-	-	-	30000	Nanogenerator	[52]

V_{OC}: Open-circuit voltage, I_{SC}:Short-circuit current, GF: Gauge factor, HGM: Hollow glass nanosphere, ADP: Ammonium dihydroge phosphate, A-CNT: Aminated carbon nanotube, Q_{tr}: Transfer charge, PEDOT:PSS: Poly(3,4-ethylenedioxythiophene) polystyrene sulfonate, FEP: Fluorinated ethylene propylene
